# Arnica/Hydroxyethyl Salicylate Combination Spray for Ankle Distortion: A Four-Arm Randomised Double-Blind Study

**DOI:** 10.1155/2011/365625

**Published:** 2011-03-07

**Authors:** Miroslav Kučera, Pavel Kolar, Milos Barna, Alexander Kučera, Marie Hladiková

**Affiliations:** ^1^Clinics for Sports Medicine and Rehabilitation, 2nd Medical Faculty of the Charles University Prague, V Úvalu 84, 15006 Prague 5, Czech Republic; ^2^Clinic for Paediatric Surgery, 2nd Medical Faculty of the Charles University Prague, V Úvalu 84, 15006 Prague 5, Czech Republic; ^3^Department for Medical Informatics, 2nd Medical Faculty of the Charles University Prague, V Úvalu 84, 15006 Prague 5, Czech Republic

## Abstract

570 patients with acute ankle joint distortion were randomized to four treatment groups: a combination spray of arnica tincture and hydroxyethyl salicylate (HES; group A, *n* = 228), arnica (B, *n* = 57), HES (C, *n* = 228), and placebo (D, *n* = 57). The medication was applied 4-5 times daily for 10 days. Efficacy was assessed on day 3-4 by evaluating pain on motion on a visual analogue scale (VAS). Pain improvement in group A was significantly superior over groups B–D (*t*-test with unadjusted baseline values, *P* < 4 × 10^−7^ and ANCOVA after adjustment, *P* < 5 × 10^−11^) and approximately corresponded to the cumulative effect of the single constituents (12.1, 7.5, and 18.7 mm VAS for A versus B, A versus C, and A versus D; 95% CI 8.0–16.2, 4.7–10.4, and 14.8–22.5 mm). The combination is justified by the additive effects of the single active constituents.

## 1. Introduction

A combination product with arnica tincture (aqueous-ethanolic extract from the flowers of *Arnica montana*) and hydroxyethyl salicylate (HES) as active constituents in the galenical formulation of a spray registered as a medicinal product has a long history of clinical use in ankle joint distortion in Germany and the Czech Republic, although there is little published clinical evidence from trials [1]. 

Topical anti-inflammatory and analgesic properties of preparations from arnica flowers are amply described in monographs [2–4]. Flavonoids and sesquiterpene lactones of the helenalin type are generally held responsible for the observed effects [5]. HES possesses local analgesic and anti-inflammatory effects, which have been confirmed in various pharmacological models [6–9], as well as in a clinical trial [10].

The local analgesic effects of the combination spray with arnica tincture and HES have been shown efficacious in a pharmacodynamic proof of concept trial in healthy volunteers using transcutaneous electric nerve stimulation for the assessment of pain threshold [11]. The study results indicated a superiority of the combination over the two single constituents. The present study has a similar design but is performed as a confirmatory clinical trial in patients. It had a twofold aim: to demonstrate the efficacy and tolerability of the combination spray against ankle joint distortion under randomized double-blind conditions versus placebo and to provide details on the contribution to efficacy of the single constituents. It was hypothesized that the effect of the combination is superior over the effect of HES or that of arnica tincture when applied as a monopreparation.

## 2. Materials and Methods

### 2.1. Trial Design

The clinical trial was designed as a prospective, randomized, reference-controlled, double-blind, and four-arm parallel group phase IV study, performed in two study centres. No changes were made after commencing the trial with respect to eligibility criteria or any other parameter.

### 2.2. Ethical Aspects

The conduct of the trial was planned and carried out in accordance with the criteria of Good Clinical Practice (GCP) and the International Conference on Harmonisation (ICH) and the ethical standards defined in the declaration of Helsinki and described by Harriss and Atkinson (2009) [12]. The trial was registered under EudraCT no. 2004-004972-35.

All patients (or, in the case of children, their legal representatives) signed an informed consent form. The trial design and the inclusion of children were approved by the local Institutional Reviewing Board (Ethics Committee) and the Czech State Institute for Drug Control (SUKL). 

### 2.3. Participants

570 patients with acute ankle joint distortion were screened and included into the study (ITT population). Patients with acute unilateral trauma of the ankle joint (lateral ankle distortion) had to present themselves within 24 hours after injury. The condition had to be sufficiently painful to require therapy. The use of anti-inflammatory or analgesic drugs, corticosteroids, and antidepressants was not permitted before and during the study, as was any kind of local treatment including physiotherapy or cold packs. Patients with fractures or complete tears of ligaments were excluded. Sports professionals and pregnant women were likewise excluded. There was no specific inclusion criterion defined regarding the severity of pain, and no stratification by severity was made.

### 2.4. Interventions

All four study preparations were based on the identically composed solution of excipients in 78% ethanol (v/v) in the presentation as a cutaneous spray (control group D). The four study preparations were produced by the same contract manufacturer (Gehrlicher Pharmazeutische Extrakte GmbH, D-82547 Eurasburg, Germany) using the same validated manufacturing method for all four medications. The basic preparation contained essential oils and camphor, which ensured that the different study preparations were not discernible by their organoleptic properties. The active medications of groups A (combination), B, and C (single substance) differed from the control preparation in group D only by the addition of arnica tincture (10 g/100 mL solution, groups A and B) and/or hydroxyethyl salicylate (HES; 3 g/100 mL, groups A and C). 

The combination preparation of group A corresponded to the medicinally authorized commercial preparations Arnidol and Sportino (study sponsor: Harras Pharma Curarina GmbH, D-81373 Munich). One stroke of the spraying device delivers 100 *μ*l of the solution. The standard dose was five strokes (500 *μ*l), delivering approximately 41.5 mg arnica tincture (groups A and B) and 12.5 mg HES (groups A and C). The intervention was applied for 10 ± 2 days.

### 2.5. Outcome Parameters

The primary outcome parameter was pain on active motion, assessed on day 3-4. Follow-up to day 10 ± 2 served for the evaluation of safety of application over the total healing time. Pain was assessed on a 100 mm nongraded visual analogue scale (VAS) directly after quickly walking a distance of approximately 10 m. 

As a secondary outcome measure, ankle swelling was measured using the figure-of-eight method [13]. The length of tape was documented in mm. Further secondary parameters included the assessment of the overall treatment effect by the physician on a five-step verbal rating scale (VRS; 1 = very  good  effect; 2 = good; 3 = moderate; 4 = minor; 5 = no  effect). For the assessment of tolerability, specific attention was given to all reports of burning and itching sensations, reddening or scaling of the skin, and urticaria or folliculitis. Other events could be described. The corresponding symptoms were rated on a four-step VRS (0 = normal; 1 = slight; 2 = moderate; 3 = severe  reaction). Moderate and severe reactions qualified as adverse events.

No changes were made to the predefined outcome measures during the study.

### 2.6. Sample Size

A sample size calculation was made based on observations from a clinical pharmacology study [11], where the increase of pain threshold was measured separately for the combination and its single constituents versus active-substance-free control by using transcutaneous electric nerve stimulation (TENS). Based on our experience with the similar efficacy of a comfrey cream preparation in the treatment of ankle joint distortion [14], we assumed a standardized difference for the arnica/HES-combination versus control of 0.75. In the TENS study, HES was approximately 1.5 times more effective than arnica tincture. Furthermore, we assumed individual standardized differences between test group and control of 0.3 and 0.45 for arnica tincture and HES, respectively. Sample sizes for the four treatment groups were calculated based on a *t*-test with an *α*-error of 0.05 and a power for every single test between 0.85 and 0.9, resulting in a total of 575 patients (*n* = 230 in groups A and C each; *n* = 65 in group B; *n* = 50 in control group D). Final sample sizes deviated to a minor extent from this calculation to allow randomisation in blocks. 

### 2.7. Randomisation and Blinding

The study preparations were manufactured and labelled according to a computer-generated random list provided by the contract manufacturer of the study medication. Concealment was ensured by fully blinding the investigators, study centres, and the statistician to the random sequence of patient allocation. 

Upon inclusion, eligible patients were double-blindly allocated by the investigators to the next available position of the randomization list in the sequence of their entry into the study, which resulted in a patient distribution ratio of 4 : 1 : 4 : 1 (group A: *n* = 228; group B: *n* = 57; group C: *n* = 228; group D: *n* = 57).

### 2.8. Statistical Methods

SPSS v.16.0 was used as the statistical software. All confirmatory analyses were made in the ITT population (all patients returning for the first visit on day 3/4). The mean decrease of pain on active motion was calculated in each group and submitted to statistical testing in the sequence of group A versus B, A versus C, and A versus D (null hypothesis: no difference between groups). Following the closed-test principle, every pair of hypotheses was tested at a two-sided significance level of 0.05, which maintains the overall significance level at 0.05. Due to considerable baseline differences in pain on motion between groups, an additional analysis of covariance (ANCOVA) adjusting for equal baseline values was performed as a descriptive parameter.

Descriptive secondary analyses including tolerability were performed in the per-protocol population at the final visit, using parametric and nonparametric statistical tests. Finally, the number needed to treat (NNT) was calculated for the comparison of group A versus D. Treatment responders were defined as patients experiencing an improvement versus baseline of at least 16 mm on the VAS in pain on active motion on day 3-4.

## 3. Results

### 3.1. Recruitment

**Figure 1:**
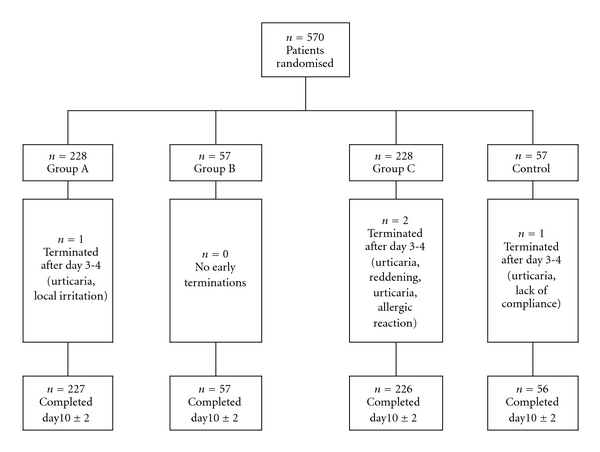
Flowchart of patient distribution to treatment groups.

The first patient was included on 31 August 2005, and the final visit of the last participant took place on 17 May 2008. A flowchart of patient distribution throughout the trial is given in Figure 1. 

570 outpatients were included and available for the ITT analysis at day 3-4. Four patients discontinued participation after the confirmatory visit (day 3-4). The inclusion and exclusion criteria were met in all cases. The demographic data are presented in Table 1.

### 3.2. Primary Efficacy Parameter: Pain on Active Motion

**Figure 2:**
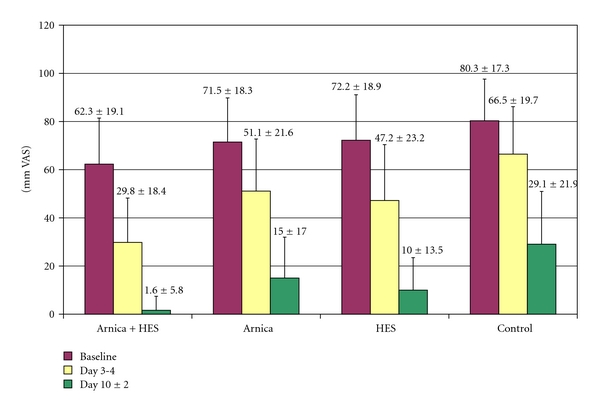
Reduction of pain on active motion (in mm VAS). Differences among groups were statistically significant (*P* ≤ 3.3 × 10^−7^, for details, see Table 2).

Pain was not equally distributed at baseline: significant differences in pain on motion were found among groups (ANOVA, *P* < .0005). Baseline pain on active motion was lowest in the combination group A ( mean = 62.31 ± 19.1 mm VAS, Figure 2). The highest baseline values were found in the control group D (mean = 80.32 ± 17.3). These baseline differences had to be taken into account in the statistical analysis. Correspondingly, the confirmatory analysis calculated with the unadjusted data (as planned in the study protocol) was compared with an analysis made with the figures adjusted for equal baseline values (Table 2). 

As expected, pain on active motion decreased over the study period in all groups (unadjusted values, Figure 2). The best results for pain reduction were found with the combination. The outcome was not different when the calculation was made with adjusted values (Table 2). 

The differences among groups were likewise statistically significant in the responder analysis (patients experiencing an improvement versus baseline of at least 16 mm on the VAS in pain on active motion on day 3-4, Table 3). The number needed to treat (NNT) was 2.5 for the combination product.

Subgroup analyses for time span between accident and presentation, origin of injury, gender, or age did not show relevant or statistically significant differences when compared with the overall outcome. The subgroup analyses generally confirmed the sequence of effects A > C > B > D (data not shown).

### 3.3. Secondary Parameter: Ankle Swelling

**Figure 3:**
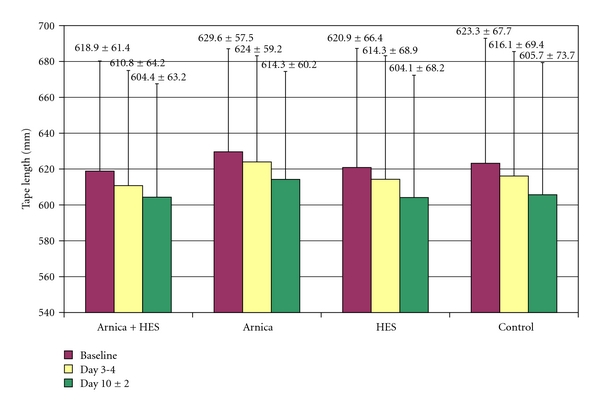
Reduction of ankle swelling (in mm tape length).

Ankle swelling decreased in all groups over time (Figure 3). The mean decrease from baseline to the confirmatory visit at day 3-4 was numerically larger in the combination group A (8.1 mm versus baseline, compared to 5.6–7.1 mm in the other groups). Healing was also quickest in group A: at day 3-4, the reduction of swelling corresponded to 57% of the overall effect at day 10 ± 2, compared to 37–41% in the other groups). The superiority of the combination versus group B was nominally significant (*P* = .047; 2-tailed *t*-test), but not versus the other groups (A versus C: *P* = .074; A versus D: *P* = .5). The study was, however, not powered to confirm differences between groups with this endpoint.

### 3.4. Global Assessment of Efficacy by the Physician

**Figure 4:**
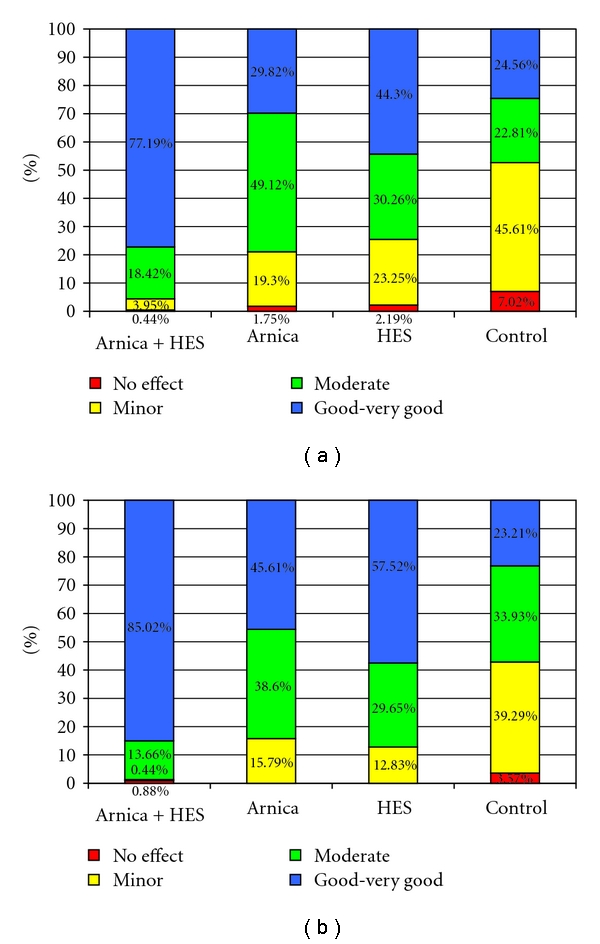
Global assessment of efficacy by the physicians at day 3-4 (visit 1, Figure 4(a)) and day 10 (visit 2, Figure 4(b)).

On the confirmatory visit at day 3-4, the global efficacy of the combination was judged to be good or very good in 77% of patients, compared to 30% in group B, 44% in group C, and 25% in the control group D. At the end of the study, these values further improved to 85% good to very good assessments in group A, 46% in group B, 58% in group C, and slightly decreased to 23% in group D (Figure 4). Statistical significance in favour of the combination was found in all group comparisons (*P* ≤ 9.1 × 10^−11^; Mann-Whitney test).

### 3.5. Safety of Application

Local intolerability reactions (burning, reddening, itching, urticaria) were observed in 4/228 patients of group A (1.75%), in 2/228 patients of group C (0.88%), and in 3/57 patients of the control group D (5.26%). No such reactions were observed in group B (*n* = 57). In the four cases, the local intolerability reaction led to discontinuation (Figure 1). There was no significant difference in incidence rate and symptom severity between the combination group and the comparative groups. 

## 4. Discussion

The group differences for pain on active motion on the VAS (a method applied in accordance with a guidance supplied by the German drug regulatory authority [15]) confirm approximately additive effects of the two single constituents, and thus provide a justification for the clinical application of the combination. As already observed in the model study with transcutaneous electric nerve stimulation [11], HES was approximately 1.5 times more effective than arnica tincture. In addition, the results for pain reduction and for the NNT must be considered clinically important. Changes in pain assessment as small as 6–8 mm on a 100 mm VAS are clearly noticeable for patients and thus clinically important [16–19]. This precondition was met for groups A–C. For the NNT, values < 4 are considered clinically important in pain reduction [20]. The calculated NNT of 2.5 for the combination is in the magnitude of the NNT found with the application of oral NSAID [21].

Local tolerability of the combination and the single constituents was better than the tolerability of placebo. The frequency of such reactions of 5% in the control group is in the range of expectations for topical drugs and specifically for ethanol-based solutions, as concentrated ethanol may irritate sensitive skin. The frequency of intolerability reactions in the Arnica and the HES groups of 1-2% is usually considered acceptable. The observation of a lower incidence rate of intolerability reactions in the combination group as compared to control might be related to the anti-inflammatory effects of the active substances which potentially contribute to the prevention of such adverse effects.

## Figures and Tables

**Table 1:** Demographic data.

Gender	
Male	358 (62.8%)
Female	212 (37.2%)

Age	30.8 ± 10.9 years (Mean ± SD)
Range	6–75 years
6–12 years	10 (1.8%)
13–17 years	36 (6.3%)
18–30 years	253 (44.4%)
31–50 years	250 (43.9%)
≥51 years	21 (3.7%)

Origin of injury	
Sports accident	63.2%
Household	18.9%
Work	11.6%
Traffic	6.0%
Other	0.4%

Time between injury and consultation	
Mean ± SD	10.1 ± 6.8 hours
Median	8 hours
Range	1–24 hours

**Table 2:** Unadjusted and adjusted intergroup differences for pain on motion versus baseline at day 3-4. A = combination; B = arnica; C = HES; D = control.

Group comparison	Relative difference (mm VAS)	95% CI	*P*-value*
Unadjusted baseline values (confirmatory analysis at day 3-4, ITT)			
A versus B	12.1	8.0–16.2	5.6 × 10^−8^
A versus C	7.5	4.7–10.4	3.3 × 10^−7^
A versus D	18.7	14.8–22.5	3.0 × 10^−16^

Adjusted baseline values (descriptive analysis at day 3-4, ITT)			
A versus B	15.2	10.9–19.4	1.4 × 10^−11^
A versus C	9.7	6.9–12.6	4.8 × 10^−11^
A versus D	24.8	20.4–29.1	3.9 × 10^−24^

*2-tailed *t*-test for equality of means for unadjusted values, ANCOVA for adjusted values.

**Table 3:** Treatment responders (ITT-population). Statistical comparisons (*χ* ^2^-test) reflect comparisons of group A with the other test groups.

Group	Responder (*n*)	%	*P *
A (Combination)	199/228	87.3	—
B (Arnica)	32/57	56.1	8.1 × 10^−8^
C (HES)	168/228	73.7	2.5 × 10^−4^
D (Control)	27/57	47.4	2.9 × 10^−11^
